# Assessment of the Artificial Intelligence–Generated Fibromyalgia Information: Beyond the Hype

**DOI:** 10.5152/ArchRheumatol.2025.11149

**Published:** 2025-09-01

**Authors:** Mert Zure, Ahmet Kıvanç Menekşeoğlu

**Affiliations:** 1 Department of Physical Medicine and Rehabilitation, University of Health Sciences İstanbul Kanuni Sultan Süleyman Training and Research Hospital, İstanbul, Türkiye; 2 Department of Physical Medicine and Rehabilitation, Mvz Berlinomed, Berlin, Germany; Corresponding author: Mert Zure ✉ mertzure@gmail.com

**Keywords:** Artificial intelligence, fibromyalgia, health misinformation, supplementary resources, trends

## Abstract

**Background/Aims:**

Individuals increasingly turn to artificial intelligence (AI) chatbots for health-related information; however, the accuracy and usability of their responses remain uncertain. This study assessed the quality, comprehensiveness, and readability of responses from 6 AI chatbots—ChatGPT-3.5, ChatGPT-4o (OpenAI), Copilot AI (Microsoft), Perplexity AI (Perplexity.AI), Gemini AI (Google), and ChatSonic AI (Writesonic)—to the most commonly searched fibromyalgia-related queries.

**Materials and Methods:**

The top 10 most frequently searched fibromyalgia-related questions from the past 2 years were retrieved from the Google Trends database. Each chatbot was queried separately, and a total of 60 responses (10 per chatbot) were assessed both qualitatively and quantitatively by 2 reviewers, focusing on content quality, accuracy, readability, and alignment with evidence-based guidelines.

**Results:**

ChatGPT-3.5 had the lowest Ensuring Quality Information for Patients score (20.6 ± 4.5), indicating very low quality information, while Gemini achieved the highest (40.5 ± 5), which was still classified as low quality. Understandability was moderate for Copilot, Gemini, and Perplexity (67.2) but lowest for ChatGPT-3.5 (43.2 ± 10.2). Actionability was weak and the misinformation assessment revealed a moderate level across all chatbots. Readability scores indicated university-level complexity, with ChatGPT-4o having the lowest Reading Ease score (11.3 ± 11.2) and Copilot the highest (30.3 ± 13.2).

**Conclusion:**

While AI chatbots provide accessible health information, their accuracy and depth vary. Gemini, Copilot, and Perplexity AI showed better quality, but citation inconsistencies, readability challenges, and misinformation risks highlight the need for refinement beyond the hype. Clinicians should guide fibromyalgia patients in critically assessing AI-generated health content. Future research should explore improvements in AI chatbot applicability for medical inquiries.

**Main Points**

- This study evaluated the accuracy, quality, and readability of responses from six popular AI chatbots to the ten most frequently searched fibromyalgia-related questions
- Overall information quality was low across all chatbots, with Gemini performing best and ChatGPT-3.5 worst, yet none reached an acceptable standard.
- While some platforms produced moderately understandable answers, all had weak actionability and contained moderate levels of misinformation.
- All chatbot responses required university-level reading skills, limiting accessibility for many patients.
- Current AI chatbots are better suited as supplementary resources rather than primary sources of medical information for fibromyalgia patients, highlighting the need for clinician-guided improvements.

## Introduction

Large language models (LLMs) are sophisticated artificial intelligence (AI) technologies that analyze enormous amounts of written information to process and produce human-like writing.^1^ These models include ChatGPT, Perplexity AI, ChatSonic AI, Copilot AI, and Gemini AI, which have all shown that they can respond to user inquiries in a logical and contextually appropriate manner.^2,3^ Large language models have quickly become popular as informational resources for the general population, including patients looking for guidance on health-related matters.^2,4^ These systems have the potential to improve patient education, fill knowledge gaps, and direct people to trustworthy resources by providing rapid access to enormous volumes of data. However, because AI-generated information is largely unregulated and constantly evolving, its quality, accuracy, and applicability in medical contexts must be carefully assessed.^1,5^

Numerous symptoms, including fatigue, cognitive impairment, and widespread pain, are hallmarks of the chronic and complex syndrome known as fibromyalgia.^6^ The subjective nature of fibromyalgia’s presentation and the lack of certain indicators make diagnosis and treatment difficult.^7^ In addition to environmental and behavioral variables, risk factors include genetic predisposition, stress, non-restorative sleep, and coexisting rheumatic disorders.^8^ The diverse treatment options—pharmacological agents, exercise programs, cognitive-behavioral therapy, and integrative medicine—prompt patients to seek clarity on risk factors, mechanisms, and treatment impacts.^6,9^

Because of this complexity, patients frequently turn to a variety of information sources, including social media networks, the internet, and increasingly, AI chatbots.^10-12^ Although these technologies can be used as additional resources, questions remain regarding the information’s accuracy, readability, and applicability.^1,3^ Evaluating the quality of AI chatbot replies to commonly searched questions regarding fibromyalgia is crucial since many patients depend on digital platforms for information about the syndrome. To the best of the authors’ knowledge, no research has examined the quality of responses provided by AI chatbots for queries related to fibromyalgia.

This study aims to evaluate the readability, accuracy, and quality of chatbot-generated answers to frequently asked fibromyalgia questions and intends to provide a foundation for guiding patients to trustworthy AI-generated information by detecting misleading information and assessing the quality of the material. Another goal is to provide information on what AI chatbots can do now and direct future plans to increase their educational value for patients and medical professionals.

## Methods

This cross-sectional study was conducted to evaluate the quality and usability of responses generated by 6 AI chatbots: ChatGPT-4o, ChatGPT-3.5 (OpenAI), Perplexity AI, ChatSonic AI, Gemini AI, and Copilot AI. The top 10 fibromyalgia-related queries found using Google Trends—a freely accessible service that monitors search term frequency over time—were used in the study. From December 3, 2022, to December 3, 2024, search patterns were the main focus of data collection. The queries are listed in Table 1.

Each query was entered into the selected AI chatbots, and responses were recorded for analysis. These responses were evaluated both qualitatively and quantitatively by 2 reviewers, focusing on content quality, accuracy, readability, and alignment with evidence-based guidelines. All queries, sourced from Google Trends’ English-language database, were entered in English to ensure consistency with the search terms analyzed. The complete verbatim responses generated by each AI chatbot for all evaluated questions are provided in Supplementary Material 1.

ChatGPT, an LLM, is designed for conversations and information retrieval, producing clear responses. However, it may provide outdated or inaccurate information due to its training data. Google Gemini, a multimodal LLM (capable of processing text and other data), excels at detailed explanations but may lack accuracy in medical topics. Perplexity AI retrieves current information online, improving accuracy with citations, though its quality depends on available web sources. Copilot AI, developed by Microsoft, combines LLM technology with web searches to summarize medical information, but it can be inconsistent if sources are limited. ChatSonic AI, built on similar technology with internet access, offers real-time responses but may produce lengthy or imprecise medical explanations.

The chatbots were used in their default settings, ensuring consistency across all interactions. A new chat session was initiated for each query to minimize bias and prevent earlier answers from influencing subsequent ones. The exact wording of each query, as identified in Google Trends, was input into the AI chatbots to ensure consistency. Disagreements were resolved by consensus. As this study did not involve human or animal participants, ethics committee approval and informed consent were not required.

### Outcomes and Measures:

The quality of chatbot responses was assessed using multiple validated instruments:

### Ensuring Quality Information for Patients:

:

The Ensuring Quality Information for Patients (EQIP) tool is a validated instrument designed to assess the quality of written health information available to patients.^13^ It serves as a checklist that evaluates various aspects of health content, including structure and identification data. The tool consists of 20 items, allowing for a comprehensive evaluation, with each item scored as yes, partly, or no. The total score is expressed as a percentage, with higher scores indicating better quality.

### Patient Education Materials Assessment Tool:

:

The Patient Education Materials Assessment Tool (PEMAT) is a systematic instrument developed by the Agency for Healthcare Research and Quality to evaluate the quality of patient education materials with scores ranging from 0% (low) to 100% (high). It focuses on 2 primary domains: understandability and actionability. Understandability assesses how easily individuals from diverse backgrounds and varying levels of health literacy can comprehend and explain the key messages of the materials. Actionability evaluates whether the materials clearly outline specific actions that patients can take based on the information.^14^

### Flesch–Kincaid Grade Level & Reading Ease Score:

Both the Flesch–Kincaid Grade Level and Flesch Reading Ease scores are commonly used to assess the readability of written content; both are based on factors like sentence length and syllable count, which provide a quantitative measure of text complexity.^15^ The Flesch Reading Ease score ranges from 0 to 100, with higher scores indicating easier-to-read text. For instance, scores above 90 are appropriate for a fifth-grade reading level, while those below 30 reflect content written at a college level.

These readability metrics are critical for evaluating whether medical content is appropriate for its intended audience, such as patients or the general public. These tools are especially useful for comparing the accessibility of various platforms and assessing the readability of online medical information, including AI chatbot responses.

### Misinformation Assessment, Word Count, and Reference Count:

Misinformation was defined as any content that contradicted or misrepresented evidence-based fibromyalgia information, as established by guidelines such as the American College of Rheumatology criteria for diagnosis and management, EULAR revised recommendations for the management of fibromyalgia, the Turkish Society of Physical Medicine and Rehabilitation guideline recommendations for the management of fibromyalgia syndrome, and more recent peer-reviewed publications.^6,9,16,17^ Two physical medicine and rehabilitation specialists assessed misinformation using a 5-point Likert scale (1 = no misinformation, 5 = high misinformation), evaluating responses for factual inaccuracies, unsupported claims, or omissions of critical information. Responses were cross-referenced with clinical guidelines and current literature to ensure consistency.

Accuracy was evaluated against these guidelines, and quality was inferred from EQIP and PEMAT scores, with consensus between reviewers ensuring standardized evaluations. Word count and reference count were also recorded to assess response length and citation use.

The median and range values of the outcomes were determined via a descriptive analysis of the data. Microsoft Excel was used to organize the data after a qualitative analysis of the responses was done to find any recurring themes or deficiencies.

### Statistical Analysis

All statistical analyses were conducted using IBM SPSS Statistics, version 25 (IBM SPSS Corp.; Armonk, NY, USA). Descriptive statistics, including means, standard deviations, medians, minimums, and maximums, were used to summarize the chatbot responses across all evaluation tools. Between-group differences in chatbot performance were evaluated using pairwise comparisons with appropriate statistical tests (e.g., Kruskal–Wallis and Mann–Whitney *U* tests) based on data distribution and sample size. A significance level of *P* < .05 was considered statistically significant.

To assess inter-rater reliability for the evaluation tools (EQIP, PEMAT-Understandability, PEMAT-Actionability, Flesch–Kincaid Grade Level, and Flesch Reading Ease score), intraclass correlation coefficients (ICCs) were calculated using a 2-way mixed-effects model with a consistency definition. Intraclass correlation coefficient values were interpreted based on established thresholds: values below 0.5 indicating poor reliability, between 0.5 and 0.75 moderate, between 0.75 and 0.9 good, and above 0.9 excellent reliability.

## Results

A total of 60 responses from 6 different AI chatbots to the top 10 fibromyalgia-related search queries were analyzed. Each query included the term “fibromyalgia” (see Appendix). On the EQIP scores, ChatGPT-3.5 had the lowest score (20.6 ± 4.5), indicating very low quality information, whereas Gemini achieved the highest score (40.5 ± 5), which is categorized as low quality. Regarding the PEMAT-Understandability scores, ChatGPT-3.5 scored the lowest (43.2 ± 10.2), while Copilot, Gemini, and Perplexity shared the highest score (67.2), rated as moderately understandable. For the PEMAT-Actionability score, ChatSonic scored the lowest (2 ± 6.3), while Copilot and Gemini achieved the highest scores (16), with all chatbots deemed weakly actionable. Misinformation, assessed using a 5-point Likert scale, was found to be moderate across all chatbots.

The length of chatbot responses ranged from 183 to 350 words, with Perplexity providing the longest responses. Flesch–Kincaid Grade Level scores ranged from 11.4 to 15.2, reflecting university-level reading complexity. The lowest Flesch Reading Ease score was found for ChatGPT-4o (11.3 ± 11.2), indicating a very difficult reading level, while Copilot achieved the highest score (30.3 ± 13.2), categorized as a difficult reading level. ChatSonic provided the fewest references (1 ± 2), whereas ChatGPT-4o and Perplexity provided the highest number of references (8) (Table 2).

In the statistical comparison between chatbots, Perplexity (*P* = .027), ChatSonic (*P* = .017), Copilot (*P* < .001), and Gemini (*P* < .001) showed significantly higher EQIP scores than ChatGPT-3.5; in PEMAT-Understandability scores, ChatGPT-4o (*P* = .014), Copilot (*P* = .001), and Gemini (*P* < .001) had higher scores than ChatGPT-3.5; there was no significant difference in PEMAT-Actionability scores; in Flesch–Kincaid Grade Level, ChatSonic had a significantly easier reading level than ChatGPT-3.5 (*P* = .024), Perplexity (*P* = .034) and ChatGPT-4o (*P* = .033); and Perplexity produced longer texts in word count compared to ChatGPT-3.5 (*P* = .017), ChatSonic (*P* = .006), and Copilot (*P* < .001) (Table 3).

Inter-rater reliability was excellent for EQIP (ICC = 0.949, 95% CI: 0.917-0.969), PEMAT-Understandability (ICC = 0.918, 95% CI: 0.866-0.950), Flesch–Kincaid Grade Level (ICC = 0.905, 95% CI: 0.847-0.942), and Flesch Reading Ease score (ICC = 0.931, 95% CI: 0.887-0.958), while moderate agreement was found for PEMAT-Actionability (ICC = 0.696, 95% CI: 0.539-0.807).

## Discussion

This study evaluated the responses generated by 6 different AI chatbots to the 10 most popular searched fibromyalgia-related queries, based on Google Trends data from the past 2 years. The results showed that the responses generally provided low-quality information, had limited actionable value, were moderately understandable yet partially inaccurate, and presented challenging reading levels.

The increasing use of AI and LLMs in healthcare highlights their potential as practical resources for patient education.^18^,^19^ These results are consistent with previous studies evaluating AI chatbots in healthcare, which have frequently reported variability in accuracy and difficulties with readability.^2,4,20^ For instance, Pan et al^2^ found that ChatGPT provided moderately accurate responses to cancer-related queries but struggled with complex topics, similar to the observations with fibromyalgia. Similarly, Parente et al^21^ reported that ChatGPT’s responses to fibromyalgia questions were generally accurate but lacked patient-oriented depth, reinforcing the conclusion that current chatbots are better suited as supplementary tools. These comparisons highlight a broader need for AI systems to improve factual consistency and accessibility across medical domains.

Comparable challenges are observed in other chronic conditions. Siu et al^20^ found that ChatGPT’s responses to colorectal cancer queries were moderately accurate but required high reading levels, limiting accessibility. Likewise, Halawani et al^19^ reported that LLMs addressing renal cancer exhibited inconsistent quality and complex language, similar to the findings with fibromyalgia.^19,^^20^ These comparisons suggest that the limitations identified in the study, such as readability issues and variable accuracy, are not unique to fibromyalgia but reflect broader challenges in AI-driven patient education across chronic diseases.

Fibromyalgia imposes a significant economic burden on healthcare systems due to frequent consultations, diagnostic procedures, and long-term treatment, compounded by indirect costs like lost productivity.^22,23^ Research emphasizes the need for more effective disease management strategies to reduce these costs and improve patient outcomes.^24^ Numerous studies have also highlighted the critical role of patient education in the management of chronic conditions such as fibromyalgia.^25^ However, it is also a well-known fact that most health information provided to patients is quickly forgotten.^26^ Therefore, innovative technologies in fibromyalgia patient education may offer a significant advantage in improving disease management.

Artificial intelligence systems have the potential to revolutionize patient education by delivering personalized, easily accessible health information.^27^ These technologies can provide interactive, real-time answers to patients’ questions. However, the findings of this study reveal that current AI models often lack quality, understandability, actionability, and readability in terms of meeting the information needs of fibromyalgia patients. This also emphasizes the need for AI systems to be trained and developed by clinicians with knowledge of the available literature. Ensuring usability is critical to maximizing the potential benefits of these tools.^28^

Consistent with the findings of this study, research on the usability of LLMs for patient information highlights challenges such as readability issues and the presence of misinformation.^18,29^ Specifically, their reliance on advanced reading levels and frequent use of medical terminology limits accessibility, hindering their ability to effectively communicate with a broader audience.

To effectively serve a diverse global patient population, AI models must also consider linguistic and cultural differences. Accurate translations and culturally sensitive content are essential to ensure accessibility and relevance. Research shows that culturally adapted interventions significantly improve patient engagement and health outcomes.^30^ Integrating these principles into AI development can expand the reach and impact of these technologies, particularly for underserved populations.

The integration of AI in patient education introduces ethical and regulatory challenges that must be addressed to prevent misinformation and ensure patient safety. Ensuring accuracy and high quality is critical to prevent harm from misinformation, while protecting patient data and confidentiality remains paramount.^31^ Regulatory frameworks should establish standardized validation protocols for AI chatbots in healthcare, requiring transparency in training data and response generation processes. Ethical guidelines must emphasize the complementary role of AI alongside professional healthcare providers, prioritizing patient safety and informed decision-making.^32^ For fibromyalgia, where misinformation can exacerbate patient confusion, such measures are particularly urgent.

A major strength of this study lies in its comprehensive, multidimensional evaluation of AI-generated fibromyalgia information using validated readability and quality assessment tools, alongside expert clinical judgment; however, certain limitations should be acknowledged. First, the assessment of misinformation was inherently subjective, relying on expert judgment rather than a standardized, validated tool. Although evaluations were performed by 2 independent clinicians with high inter-rater agreement, the potential for interpretation bias remains. Second, while the accuracy and relevance of chatbot-provided references are examined, a systematic verification of each citation’s authenticity and content was beyond the scope of this study. Third, the analysis was based on a limited set of frequently asked questions, which may not fully capture the breadth and variability of information patients seek regarding fibromyalgia. Lastly, the fast-evolving nature of chatbot algorithms poses challenges to reproducibility, as the models are continuously updated and refined, potentially leading to different outputs over time. Future research should address these issues through broader question sets, inclusion of patient feedback, and dynamic tracking of AI-generated content across different time points.

This study evaluated the state-of-the-art of current software developments in informing patients diagnosed with fibromyalgia and revealed significant gaps in the use of chatbots. However, for future work, it is recommended to develop AI models by integrating comprehensive datasets and improving natural language processing. To improve AI chatbot utility, developers should prioritize clinician-led training to align outputs with evidence-based guidelines, enhance citation transparency to verify sources, and design responses for lower reading levels to suit diverse patient populations. Involving patients in the development process can help tailor these tools to meet their specific needs. Future research should explore patient-centered co-design processes, where fibromyalgia patients contribute to refining chatbot functionalities, and longitudinal studies to assess the impact of AI-based education on health outcomes.

The use of AI chatbots to obtain medical information is becoming increasingly widespread. Although LLMs generally provide accurate information about fibromyalgia, the quality, understandability, actionability, and readability of their outputs remain insufficient. These shortcomings indicate that AI chatbots are currently better suited as supplementary tools rather than primary sources of medical information. The findings emphasize the need for clinician involvement in the development and validation of these tools to ensure their alignment with the standards of comprehensive healthcare.

## Figures and Tables

**Table 1.** Top Fibromyalgia-Related Search Queries from December 3, 2022, to December 3, 2024, According to Google Trends

1	Fibromyalgia symptoms
2	Is fibromyalgia
3	Fibromyalgia pain
4	What is fibromyalgia
5	Fibromyalgia cause
6	Fibromyalgia treatment
7	Symptoms of fibromyalgia
8	Fibromyalgia syndrome
9	Fibromyalgia arthritis
10	Fibromyalgia test

**Table 2.** Evaluation of Artificial Intelligence Chatbot Responses to Search Queries Relating to Fibromyalgia

Measure	Mean (SD) (Min-Max)					
	Copilot	ChatGPT-3.5	ChatGPT-4o	Chatsonic	Gemini	Perplexity
EQIP	38.2 (6) (32.1-44.4)	20.6 (4.5) (15.6-28.1)	29.9 (6) (18.8-38)	35 (3) (30-38.9)	40.5 (5) (32.1-50)	33.8 (5.5) (23-42.3)
PEMAT-Understandability	67.2 (7.5) (54.5-75)	43.2 (10.2) (30-58.3)	64.4 (8) (44.4-70)	56.8 (6.8) (44.4-68.3)	67.2 (6.8) (50-75)	67.2 (6.8) (46.7-70)
PEMAT-Actionability	16 (8,4) (0-20)	4 (8.4) (0-20)	4 (8.4) (0-20)	2 (6,3) (0-20)	16 (8,4) (0-20)	10 (10.5) (0-20)
Misinformation	2 (1) (1-3)	3 (1) (2-4)	2 (1) (1-3)	3 (1) (2-3)	2 (1) (1-3)	3 (1) (1-5)
Flesch–Kincaid Grade Level	12.9 (1.9) (9-15.9)	15 (3) (10.1-18.2)	15.2 (3.7) (10-21.8)	11.4 (1.4) (8.4-13.4)	12 (1.3) (10.3-14.7)	14.3 (1.3) (12.4-17)
Flesch Reading Ease Score	30.3 (13.2) (11.6-51.2)	14.5 (11.6) (0.4-37.6)	11.3 (11.2) (0-37.9)	27.9 (8,3) (18.2-47.3)	29.9 (9.2) (9.4-34.3)	17 (8.1) (3.6-31.6)
Word count	183 (54) (81-273)	240 (100) (131-444)	298 (66) (157-373)	226 (56) (124-312)	262 (53) (170-312)	350 (45) (289-452)
Reference number	2 (2) (0-4)	6 (1) (5-8)	8 (3) (5-13)	1 (2) (0-5)	3 (4) (0-8)	8 (1) (6-9)

EQIP, Ensuring Quality Information for Patients; Max, Maximum; Min, Minimum; PEMAT, Patient Education Material Assessment Tool.

**Table 3.** Evaluating the Performance of Artificial Intelligence Chatbots

Chatbots	EQIP	PEMAT-Understandability	PEMAT-Actionability	Misinformation	Flesch–Kincaid Grade Level	Flesch Reading Ease Score	Word Count	Reference Number
ChatGPT-3.5-ChatGPT4o	0.890	**0.014**	1	**0.014**	1	1	0.844	1
ChatGPT-3.5-Perplexity	**0.027**	1	1	1	1	1	**0.017**	1
ChatGPT-3.5-Chatsonic	**0.017**	1	1	1	**0.024**	0.175	1	**0.033**
ChatGPT-3.5-Copilot	**<0.001**	**0.001**	0.104	0.093	1	0.143	1	0.097
ChatGPT-3.5-Gemini	**<0.001**	**<0.001**	0.104	0.093	0.145	0.052	1	1
ChatGPT4o-Perplexity	1	1	1	0.214	1	1	1	1
ChatGPT4o-Chatsonic	1	1	1	0.056	**0.033**	**0.033**	0.434	**<0.001**
ChatGPT4o-Copilot	0.121	1	0.104	1	1	**0.026**	**0.015**	**0.001**
ChatGPT4o-Gemini	**0.01**	1	0.104	1	0.187	**0.008**	1	**0.026**
Perplexity-Chatsonic	1	1	1	1	**0.034**	0.635	**0.006**	**<0.001**
Perplexity-Copilot	1	0.124	1	0.910	1	0.535	**<0.001**	**0.001**
Perplexity-Gemini	0.438	0.075	1	0.910	0.191	0.228	0.148	**0.043**
Chatsonic-Copilot	1	0.281	0.056	0.299	1	1	1	1
Chatsonic-Gemini	0.612	0.177	0.056	0.299	1	1	1	1
Copilot-Gemini	1	1	1	1	1	1	0.587	1

EQIP, Ensuring Quality Information for Patients; PEMAT, Patient Education Material Assessment Tool.

## Data Availability

The data that support the findings of this study are available on request from the corresponding author.
